# Introducing *PLoS Clinical Trials*: A different approach to publishing trial results

**DOI:** 10.1371/journal.pctr.0010008

**Published:** 2006-05-19

**Authors:** Emma Veitch

Welcome to *PLoS Clinical Trials*. This new journal is devoted to providing an unbiased, peer-reviewed forum for trial results in all fields of medicine and public health. But why is a new journal needed?

In the world of clinical trials, the current publishing system does not work in the best interests of patients, clinicians, or health policymakers. All these groups of people should be able to base their decisions on good-quality systematic overviews of all the available evidence. Thorough systematic review requires access to and careful evaluation of all the primary research studies that address the question of interest, and robust mechanisms are therefore needed for unbiased dissemination of the results of clinical research. However, bias is known to exert an effect at virtually every stage, from study concept and design [1] to write-up and publication [2,3]. In an Essay published as part of the first group of papers in *PLoS Clinical Trials,* David Korn and Susan Ehringhaus [4] identify an urgent need for higher standards, and discuss why in particular all those engaged in trial sponsorship and conduct must work toward universal disclosure of study results. Current efforts toward universal prospective registration of trials [5] will contribute significantly toward this goal. Registration ensures that trials are publicly known from the start, encouraging full and transparent reporting.

So what is *PLoS Clinical Trials* doing to achieve the goal of reducing bias? The journal is a crucial step toward making peer review and publication more impartial. Reviewers and academic editors for *PLoS Clinical Trials* are asked to focus on whether a trial's methods are appropriate and ethical, the analyses sound, and the interpretation accurate. Provided that these things are done, the trial will be published, irrespective of whether a prespecified analysis adds truly novel results, refutes something that was previously thought to be the case, or provides confirmatory data.

The results are reported in a structured format based on the consolidated standards of reporting trials (CONSORT) guidelines [6], making it easy for readers to understand what was done and what the results mean. Editorial summaries written by editorial staff, based on the comments of academic editors and expert reviewers, will accompany each paper, and will provide an important mechanism for describing without frills what was found and what the results add to the evidence. And, of course, open-access publication ensures that trial results will be available immediately to all for reading, reuse, or reanalysis through the Web site, as well as PubMed Central. *PLoS Clinical Trials* also provides investigators and funders with a peer-reviewed journal publication—a just reward for the huge investment that can be involved in running trials.

In the short period, since *PLoS Clinical Trials* was announced, submissions have arrived from around the world, including Australia, Belgium, The Gambia, Germany, Mozambique, the Netherlands, Spain, Uganda, the United Kingdom, and the United States. The journal's scope encompasses trials testing the effects of all types of interventions related to health care. So far, submissions to the journal have reflected this diversity, and have included not only pharmaceutical studies but also trials testing experimental vaccines, surgical procedures, and complex and educational interventions. The valuable guidance and support of the *PLoS Clinical Trials* advisory board, editorial board, and statisticians have helped to shape the journal's launch, but we intend to continue evolving in response to feedback from you and the broader community.

Perhaps you agree with the proposals put forward by Smith and Roberts [7], that the future of trials publishing should not involve medical journals. Instead, they propose that trial protocols and complete datasets should be published on the Web. There, the community can comment on trial design and contribute toward appropriate analysis of the results. By running only prespecified analyses, and by removing interpretation, except in the context of the overall evidence, spin and bias of individual trial results are eliminated. The Global Trial Bank (GTB) [8] will offer such a solution for the future, and *PLoS Clinical Trials* is partnering with GTB to make universal access to computable trial data a reality.

So, if you share our vision for an unbiased clinical research literature, we invite you to contribute to *PLoS Clinical Trials*. You can comment on the articles that we've published and send your presubmission inquiries via the Web site at http://www.plosclinicaltrials.org. But we also welcome comments and ideas—however radical—for the journal's future. We look forward to hearing from you at plosclinicaltrials@plos.org.
